# Our Life Is a Rollercoaster! A Qualitative Phenomenological Study Exploring the Impact of IBD on Family Members

**DOI:** 10.1093/ibd/izae028

**Published:** 2024-02-28

**Authors:** Parichat Thapwong, Christine Norton, Emma Rowland, Wladyslawa Czuber-Dochan

**Affiliations:** Florence Nightingale Faculty of Nursing, Midwifery and Palliative Care, King’s College London, James Clerk Maxwell Building, 57 Waterloo Road, London, United Kingdom; Faculty of Nursing, Chiang Mai University, Chiang Mai, Thailand; Florence Nightingale Faculty of Nursing, Midwifery and Palliative Care, King’s College London, James Clerk Maxwell Building, 57 Waterloo Road, London, United Kingdom; Florence Nightingale Faculty of Nursing, Midwifery and Palliative Care, King’s College London, James Clerk Maxwell Building, 57 Waterloo Road, London, United Kingdom; Florence Nightingale Faculty of Nursing, Midwifery and Palliative Care, King’s College London, James Clerk Maxwell Building, 57 Waterloo Road, London, United Kingdom

**Keywords:** Crohn’s disease, family members, inflammatory bowel diseases, quality of life, ulcerative colitis

## Abstract

**Background:**

Inflammatory bowel disease (IBD) significantly impacts patients and their families. To provide support, understanding the effects on the wider family is crucial. However, limited research exists on the impact of IBD on family members of adults diagnosed with IBD. This study addresses this knowledge gap.

**Methods:**

Underpinned by interpretive phenomenology, this study used in-depth, semi-structured online interviews to explore relatives’ experiences. Interviews were audio-recorded and transcribed verbatim. Data were analyzed using reflexive thematic analysis.

**Results:**

Forty-three purposively selected interviewees comprising 17 people with IBD and 26 family members (parents, children, siblings, and partners) revealed 3 main themes: (1) “life is a rollercoaster,” (2) “there have been a lot of bridges to cross along the way,” and (3) “my life would be better if…” Participants highlighted that IBD has both positive and negative impacts on family members in terms of emotional well-being, relationship, roles and responsibilities, day-to-day burden, and sibling suffering. Some employed adaptive coping strategies such as creating social networks and open communication, while others relied on maladaptive coping strategies, such as avoidance and alcohol abuse. Family members expressed the need for proactive communication, information, and support from healthcare professionals.

**Conclusions:**

IBD affects the emotional and psychosocial well-being of family members, eliciting both adaptive and maladaptive coping strategies. Healthcare professionals need to adopt a holistic approach to managing IBD that considers the psychosocial and emotional challenges faced by individuals and their families.

**Key Messages**

**What is already known?**

- Inflammatory bowel disease (IBD) significantly affects the quality of life of both patients and their family caregivers.

**What is new here?**

- The impact of IBD extends beyond the primary caregivers and affects the broader family unit in terms of psychosocial and emotional well-being.
- Family members of individuals with IBD require guidance on coping strategies and interventions to support their psychological, social, and emotional well-being.

**How can this study help patient care?**

- Understanding how IBD affects families, their coping strategies, and the necessary support empowers families to actively participate in care, resulting in holistic support and enhanced patient outcomes.

## Introduction

Inflammatory bowel disease (IBD) is a chronic, progressive, and lifelong disease involving relapsing and remitting symptoms. IBD includes ulcerative colitis (UC) and Crohn’s disease (CD).^1,2^ In 2017, over 6.8 million people were experiencing IBD globally.^3^ It is estimated that in the United Kingdom, the prevalence of UC is nearly 600 cases per 100 000 people and the prevalence of CD is 400 cases per 100 000 people.^4^ IBD can affect people of any age, and it exerts significant impacts on working-age groups, leading to substantial personal, emotional, societal, and economic challenges.^5,6^ Common IBD symptoms include frequent, urgent, and profuse diarrhea, rectal bleeding, abdominal pain, malnutrition, and fatigue.^7^ IBD can place severe restrictions on a person’s daily life, including scheduling frequent hospital appointments, adherence to dietary restrictions, various lifestyle changes, and a need to stay close to a toilet. These can all have a significant impact on individuals’ psychological well-being and their ability to work or attend school, perform parental duties or other personal relationship roles, or spend time on social and leisure activities.^8,9^

The severity of IBD symptoms may increase individuals’ reliance on their family to act as caregivers, creating additional psychosocial and emotional burdens for both patients and family members.^10^ The direct impact of the disease, coupled with the burden of providing caring support, means that IBD can significantly affect a family’s quality of life (QoL). Quantitative surveys of the impact of IBD on adult patients’ primary caregivers have indicated that many experience a high level of burden and low QoL but have not considered the experiences of other family members.^11,12^

A systematic review synthesizing 33 studies exploring the impact of IBD on family reported a significant negative effect on family members’ emotional well-being, relationships with the patient, social life, work and finances, and leisure time and travel.^13^ However, most of the data were from cross-sectional surveys focused on the impact of IBD on the families of pediatric populations. Only 3 qualitative studies have been found that focused on family burden in adults with IBD, which demonstrated that family caregivers faced psychosocial challenges.^14-16^

Previous studies have tended to focus specifically on the primary caregivers, rather than on the impact on the broader family unit. Given the potential impact of IBD on all family members (eg, parents, children, partners, siblings), this needs to be addressed. While standards for IBD care in the United Kingdom emphasize the need for more accessible services for people with IBD,^17^ research suggests that members of their families do not have adequate support.^13,14^ The well-being of the family may also have a direct outcome on the health of the person with IBD.^18^ It is therefore important to gain understanding of how living with a person with IBD affects families, to facilitate the development of evidence-based interventions to support families and enhance their well-being and that of the person with IBD.

## Methods

### Aims

We sought to (1) explore the experiences of family members who have an adult relative with IBD, (2) examine how families manage any impact of the disorder, and (3) identify how to optimally support family members who have a relative with IBD.

### Design

This exploratory qualitative study was guided by interpretive phenomenology, also referred to as hermeneutics, defined by Heidegger in the 1960s as the study and interpretation of human behavior. Heidegger believed that human beings are inseparable from their world: being human is being there in the world, while being in the world allows for new knowledge and understanding to evolve, which leads to the interpretation of the new experience.^19^ Understanding the world of the participants is essential for interpreting their experiences. This philosophical framework focuses on a deeper understanding of the lived experiences of individuals and interpreting the meanings they attribute to these experiences,^20^ making it well suited for exploring the multifaceted effects of IBD on family members.

At its core, hermeneutic phenomenology encourages a deep engagement with participants’ narratives, urging researchers to undertake a reflective and interpretive analysis. In this regard, researchers use their existing knowledge and broader understanding of the subject matter in the research process. In alignment with this principle, we possess a foundation of knowledge gleaned from prior research endeavors focused on the impact of IBD on family members. This collective expertise served as a robust foundation for a comprehensive and insightful exploration of the phenomenon.

This study also incorporated Bowen’s family systems theory (FST) to explore the impact of IBD on family members.^21^ Proponents of FST view a family as an emotionally interconnected unit, where the actions of one member affect the entire family. Each individual’s well-being influences the overall family dynamic. In FST, the importance of considering the family as a whole in understanding chronic disease effects is highlighted, as family interactions significantly shape individual experiences and management.^22^ This theory provides a comprehensive framework to explore the impact of IBD on family dynamics, illuminating emotional, relational, and psychological effects. By employing this perspective, this study aimed to offer a holistic understanding of the broader consequences of IBD within families.

### Participants

Participants were recruited via the website and social media channels of 2 charities (Crohn’s and Colitis UK and Bowel Research UK) and the research volunteer recruitment portal at King’s College London. The inclusion criteria for patient participants were (1) being clinically diagnosed with IBD (either CD or UC) for at least 1 year, (2) being 16 years of age or older, (3) being able to speak English well enough to take part in an interview, and (4) having had a relative willing to take part in this study.

Inclusion criteria for family members were (1) being a parent, spouse, child, or sibling of a person with IBD who was taking part in the study; (2) living or having lived in the same house with the person with IBD (no time frame specified); (3) being 16 years of age or older; and (4) the ability to speak English well enough to take part in an interview.

A total of 151 people requested the study information sheet. Fifty-seven people (24 people with IBD, 33 family members) were deemed eligible. From this pool, purposive sampling was used to capture participants with different types of disease (CD/UC), sex (male, female), and ethnicity. Family members were also sampled purposively to capture participants with different relationships with patients (eg, partners, parents, children, siblings). For each patient, between 1 and 3 family members of their family were included.

### Data Collection

In-depth online interviews were conducted via MS Teams, Zoom, or telephone between December 2021 and June 2022. People with IBD and family members were interviewed separately. A semi-structured interview guide was developed via a literature review and Patient and Public Involvement consultation (see Tables 1 and 2, Supplementary Data Content). The interviews were individually conducted by both the first and fourth authors, who are experienced in conducting in-depth interviews. The duration of interviews ranged from 35 to 85 minutes (with a mean of 57 minutes), totaling approximately 41 hours and 48 minutes of interviews. There were no prior relationships between interviewers and participants. The audio recordings were transcribed verbatim by a professional transcriber and were anonymized before analysis. The interviewers recorded field notes immediately after each interview.

**Table 1.** Patients’ characteristics

**Age, y**	**39 (20-84)**
**Sex**	
Male	4
Female	13
**Confirmed diagnosis of IBD**	
CD	10
UC	7
**Time since IBD diagnosis, y**	**15 (1-53)**
**Ethnicity**	
White—British	15
White—any other White background	1
Asian Pakistani	1
**Education level**	
School level qualifications	3
Advanced school level qualifications	1
University degree	10
Postgraduate degree	2
Other professional qualifications	1
**Relationship status**	
Married/living with partner	16
Single	1
**Number of children**	
0	11
1	2
2	4
**Current employment status**	
Employed or self-employed full-time	7
Employed or self-employed part-time	3
Full- or part-time education	5
Retired	2
**Previous surgery for IBD**	
Yes	8
No	9
**Number of surgeries**	0-3
**Current stoma**	
Yes	2
No	15
**Number of IBD flares in the last 2 y**	0-5
**Severity of symptom during last flare**	
None	1
Mild	3
Moderate	7
Severe	6

Values are mean (range), n, or range.

Abbreviations: CD, Crohn’s disease; IBD, inflammatory bowel disease; UC, ulcerative colitis.

**Table 2.** Relative’s characteristics

**Age, y**	**46 (19-79)**
**Sex**	
Male	13
Female	13
**Relationship to the person with IBD**	
Father	3
Mother	8
Partner	6
Sibling	4
Children	5
**Ethnicity**	
Asian or Asian British—Pakistani	3
White—British	20
Mixed—any other mixed background	1
White—any other White background	2
**Education level**	
Vocational qualifications	2
School level qualifications	6
Advanced School level qualifications	2
University degree	15
Postgraduate degree	1
**Current employment status**	
Employed or self-employed full-time	12
Employed self-employed part-time	3
Full- or part-time education	4
Retired	7

Values are mean (range) or n.

Abbreviation: IBD, inflammatory bowel disease.

### Data Analysis

The theoretically flexible method of reflexive thematic analysis was used, enabling the development, analysis, and interpretation of patterns of meaning across a dataset.^23^ NVivo version 12 (QSR International) was used to manage the data. All 4 authors were involved in the data analysis process. In the first stage, we thoroughly familiarized ourselves with the data by repeatedly reading the transcripts (12 transcripts were read by P.T., and 4 transcripts by each coauthor). During this process, we made notes of initial trends, identified interesting passages, and documented our thoughts and feelings related to both the data and analysis process.

Then, we each coded interviews using both inductive and deductive coding processes. Inductive coding involved codes that developed organically from data, such as social restriction and loneliness. Deductive coding involved codes derived from applying prior knowledge, personal experience, and pre-existing concepts from FST to the data,^24^ such as differentiation of self and emotional cutting off. By employing these coding approaches simultaneously, the aim was to construct both the latent and semantic meanings embedded within the data.^23^ We met multiple times to discuss our codes so as to achieve greater depth in meaning and alternative interpretations.

Following these discussions, P.T. developed an initial code list, arranged in alphabetical order and containing codes generated during the process of coding, which prevented new duplicate codes being created during subsequent analysis. Continued data familiarization and the first iteration of the coding process was completed with the remaining transcripts. All 4 authors came together to further discuss the data, and the codes were then merged and collapsed in which we shared the same underlying meaning to create a second iteration of codes.

This was further refined by E.R. in a third iteration of code development. Moving into the fourth iteration, P.T. then used Miro, a digital whiteboard platform, to visually merge the codes to build themes and subthemes that would enable the construction of a coherent narrative of the data through thematic mapping.^23^ The thematic map was shared with the other authors, the themes and subthemes were discussed, and the content and the name of the themes were modified and confirmed.

### Trustworthiness of Data

To ensure the rigor of the research, the team considered key components of trustworthiness, namely credibility, dependability, confirmability, and transferability.^25^ In this research process, we used prolonged engagement with data, field notes, reflexive journaling, and a description of the audit trail, and we documented reasons for theoretical, methodological, and analytical choices throughout the study. We ensured that reflexivity was integrated throughout the research process by being conscious of the potential impact of professional roles and personal backgrounds on data collection and analysis. We all are experienced in qualitative research. Both interviewers (ie, P.T. and W.C.-D.) have a professional background in nursing and a research interest in IBD. C.N. is an academic with experience in IBD research. E.R. is emotional health geographer with research experience in the psychosocial and emotional impact of long-term conditions on family members and their relationships.

### Ethical Considerations

Ethical approval for this study was granted by the King’s College London research ethics committee (reference number: HR/DP-21/22-26069). As all the participants were interviewed remotely, verbal informed consent was obtained and audio-recorded at the beginning of each interview. All study data were kept confidential and secure in line with university policy, and the General Data Protection Regulation.

### Results

In total, 43 participants (17 people diagnosed with IBD and 26 of their family members) were interviewed. The sociodemographic and health data of the participants are shown in Tables 1 and 2.

**Figure 1.**
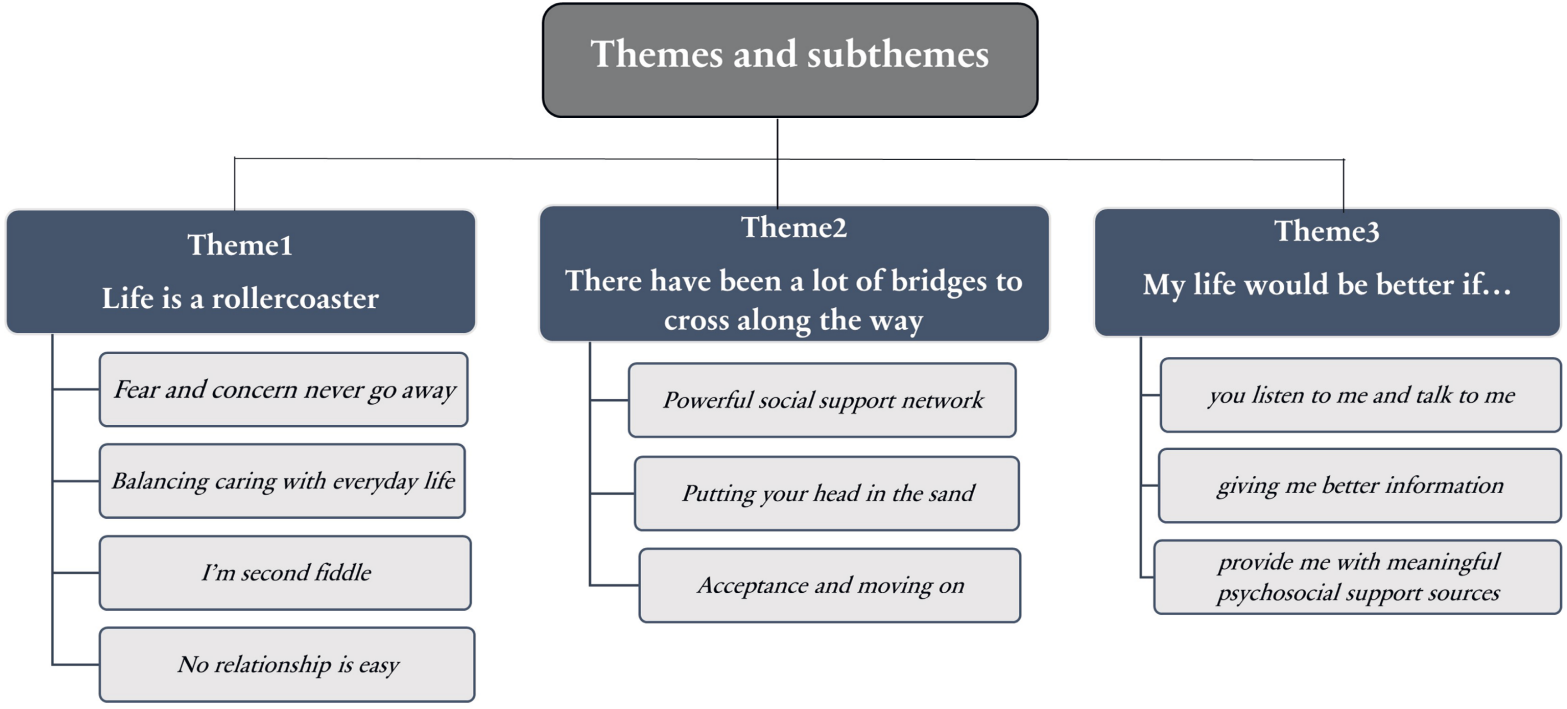
Study themes and subthemes.

Through reflexive thematic analysis, we developed 3 main themes and accompanying subthemes that described the data, as shown in Figure 1: (1) “life is a rollercoaster,” (2) “there have been a lot of bridges to cross along the way,” and (3) “my life would be better if…”

These themes are described subsequently, illustrated by verbatim quotations in the text, with additional supporting excerpts from interviews in Table 3.

**Table 3.** Themes, subthemes, and example quotes reflecting participants’ experience on the impact of IBD on family members

Theme	Subtheme	Example quotes
**Life is a rollercoaster**	**Fear and concern never go away**	#1. “It was a nightmare of a time, an absolutely nightmare and it has been this past year has been awful. […] It’s a rollercoaster of emotions. It’s anger, it’s frustration, it’s fear, it’s anxiety, it’s not fair, why is it happening to my daughter, what did she do wrong, it’s just horrible absolutely horrible.” (Family11, FM19, Mother)
		#2. “That’s scary because I don’t know how that’s going to affect her long term. Will that reduce her life expectancy? She might want to have children—does that affect her fertility?” (Family11, FM18, Father)
		#3. “I think we are a bit more worried and fearful than she is. I don’t know if that’s because you see it from an outsider’s perspective. You can recognize how bad someone is getting and they can.” (Family6, FM9, Daughter)
	**Balancing caring with everyday life**	#4. “I was just tired…I was going to the hospital and taking him home and then going into work. If I’d had a morning appointment, I’d work through my lunch hour, so I worked five or six hours straight.” (Family4, FM7, Mother).
		#5. “She facetimes me because she’s sad and she needs to talk, so I stop working and I talk […] So I feel like I’m working more hours, a lot more hours but I’m probably not achieving any more.” (Family11, FM19, Mother)
		#6. “If my daughter is not fit to work properly, it can affect her. She has a house to run so she has got bills to pay. We [parents] try to support our daughter the best way we can. […] We are there for our daughter and son both financially. Nobody seems to care, especially the Government.” (Family12, FM21, Mother)
	**I’m second fiddle**	#7. “It is if she needed to use the toilet frequently and it could be embarrassing for her as well. That’s the thing I wasn’t just sitting there thinking of myself there it could be embarrassing for her, it could be somebody she knew that I was bringing over so for her to then have to keep going to the toilet as a teenage girl you don’t want people from the same school knowing that do you.” (Family1, FM2, Brother)
		#8. “My son was completely ignored […] He was missing out and he spent a lot of time on his own because I was at the hospital with her. It was very, very difficult.” (Family1, FM1, Mother)
		#9. “But there was always that element to it where I thought I’m second fiddle…I got my degree, party time and all that it was good—but then my sister gets her degree and it’s while having Crohn’s.” (Family1, FM2, Brother)
	**No relationship is easy**	#10. “I would look back and say it was actually a good thing for us and it taught us all a thing or two about life, the universe and everything. It’s definitely made us all more aware of illness and disability and it’s made us all tougher.” (Family1, FM1, Mother)
		#11. “It did impact on our relationship for quite a while. Resentfulness on both sides […] he was resentful of me because he thought I was trying to mother him (diet and medication control) and I suppose I was resentful of him because of the way he was.” (Family4, FM7, Mother)
		#12. “For me I think we have a different relationship now to what we had and that’s not because, I’m not saying it’s because of our daughter’s disease but I think it’s my wife’s reaction to our daughter’s disease and how she worries so much.” (Family 11, FM18, Father)
**There have been a lot of bridges to cross along the way**	**Powerful social support network**	#13. “Her [partner’s] mum and her sister are amazing. They look after my son quite a lot, they take him once or twice a week. They know everything that’s going on so they’re just a phone call away if we need them.” (Family17, PT17)
		#14. “I quite often look at these, I’m linked into the ulcerative colitis and different websites on Facebook just to get knowledge really and understanding and Crohn’s & Colitis society.” (Family7, FM10, Mother)
		#15. “At the time I worked part-time and where I worked were extremely good at giving me time off if I needed to take her to the hospital, for doctor’s appointments.” (Family14, FM23, Mother)
	**Putting your head in the sand**	#16. “I think I was in college when there was a really close call with her and I just went to college, went about my normal day, did my homework, tried to keep busy and not think about it.” (Family1, FM2, Brother)
		#17. “At that time my then wife started becoming an alcoholic and this was just one more pressure upon her when these flare-ups were going on.” (Family3, FM6, Father)
	**Acceptance and moving on**	#18. “On the airplane there’s always a toilet plus my wife seems to be able to manage that situation very well. She takes medication to try and stop anything like that happening, Imodium, and that sort of thing seems to work quite well if she’s traveling.” (Family5, FM8, Partner)
		#19. “My sister, she had it so bad, so, so terribly bad and yet she now has really quite a nice life. I think that that’s a good thing to be focusing on. Looking at the other side and that there is a future and it’s a shiny one.” (Family15, FM24, Sister)
		#20 “I think certainly for me and my daughter…the exercise side really, really helps and particularly if you are out for a long walk you can chat about things when you are walking as well. So, exercise plays a big part in […] the mental recovery as well as the physical for her and for me too.” (Family14, FM23, Mother)
**My life would be better if…**	**You listen to me and talk to me**	#21 “I think that would have lifted a massive [weight off my] shoulders—and would make you not so much during the day be there like thinking oh my God what if something is happening and she’s there on her own, no-one is there and there’s no way you can talk to them [HCPs].” (Family6, FM9, Daughter)
		#22 “It would have been nice to have been able to sit with somebody like an IBD nurse to say right and then I could have just said, it’s all right saying you can do this, you can ring the helpline.” (Family7, FM10, Mother)
		#23 “It would be good to have an opportunity to speak to the IBD nurse as family because it’s a family issue. It’s patient confidentiality isn’t it—you don’t really get involved.” (Family17, FM26, Partner)
	**Giving me better information**	#24. “I personally would like to understand more about sex…I think there’s probably an intervention somewhere which says ‘sex, stomas and whatever’—yet there’s something which says to partners or long-term partners if you have a partner with Crohn’s read this, talk to this.” (Family16, FM25, Partner)
		#25. “I just want to know how I can better support her. It’s really hard to find out how I can do that because information is so different out there [online source].” (Family 8, FM11, Father)
		#26 “The first thing you do when you are a parent, and you are faced with something like that is start Googling which is absolutely terrifying because you hear all the horror stories then. I don’t think you get a particularly balanced view when you start Googling these things.” (Family11, FM19, Mother)
	**Provide me with meaningful psychosocial support sources**	#27 “It would have been nice to have had some financial support whilst she was going through the worst bit at the beginning, so we could have taken time off as a family to focus on getting her through this instead of trying to juggle work and everything else.” (Family11, FM19, Mother)
		#28 “There are just not enough support groups out there for people to tell their experience and their day-to-day living and their life or what they go through because there’s just not that support network out there.” (Family12, FM21, Mother)

Participants are identified by family, FM, or PT number, and relationship to the person with IBD given in parentheses after each quote.

Abbreviations: FM, family member; HCP, healthcare professional; IBD, inflammatory bowel disease; PT, patient.

### Theme 1: Life Is a Rollercoaster

There were frequent and unpredictable changes to the physical and emotional health of the person with IBD. This instability in health for the person with IBD had an impact on family members’ lives and relationships with each other, creating fluctuating emotions:

“It was a rollercoaster, it was up, down, up, down. She’d be OK and then she wouldn’t be OK and then she was nearly dying, then she was OK again for a bit. It’s distressing because it’s a member of your family who may die.” (Family15, family member 24 [FM24], Sister)

#### Subtheme 1: fear and concern never go away

Having a family member with IBD clearly had an impact on the mental and emotional well-being of many of the participants. They expressed feelings of shock, fear, relief, anxiety, and upset throughout the lifetime of the diagnosis, which tended to come and go depending on disease activity (eg, Table 3, quote 1). Many families reported inadequate information about IBD from professionals and that this had compounded their anxiety. Uncertainty and confusion about the impact of IBD on their relative’s future, education, work, and relationships led to high levels of stress.

Given the possible genetic component of IBD, some sibling participants were also concerned about whether they themselves would develop the condition. Others were worried about serious IBD flare-ups, and about their relative dying early or not living a full life (eg, Table 3, quote 2). Such anxiety affected family members through loss of appetite and sleep disturbances. Participants explained how they felt they were sometimes more anxious about the condition than their relative who experienced it (eg, Table 3, quote 3). However, despite these high levels of anxiety, few participants reported obtaining support or having treatment to improve their psychological well-being, and indeed many felt guilty about focusing on themselves given their relative’s health.

#### Subtheme 2: balancing caring with everyday life

Parents related the burden of having to take care of a person with IBD during a disease flare-up, alongside caring for other dependents, working, and running a household. This was an immense strain when they were not supported, for example, in single-parent families. Parents of people with IBD talked about the pressure and accompanying exhaustion associated with their caring role (eg, Table 3, quote 4). Family members felt called upon to provide emotional as well as tangible support, which often created a tension between attending to caring duties and being productive in their own lives (eg, Table 3, quote 5).

Caregiving responsibilities also created financial challenges, as relatives had to adapt their employment obligations, such as reducing their hours, taking time off from work, or leaving their jobs. Meanwhile, direct costs of IBD included paying for medication and transport and meeting expensive dietary requirements were sometimes significant challenges. Some participants tried to apply for extra funding, but this process itself often caused more stress, and parents often felt compelled to step in and assist their children financially (eg, Table 3, quote 6).

#### Subtheme 3: I’m second fiddle

Clear limitations were placed on the entire family, with the need to adhere to dietary restrictions affecting family meals, and the need to consider things such as toilet access, which limited traveling or could sometimes “ruin” family holidays. All members of the family had to adjust their social lives, and siblings reported that they refrained from inviting friends to their home to protect their ill siblings, and avoid creating situations that could potentially embarrass the patient (eg, Table 3, quote 7).

Families commonly reported the huge amount of time taken up by hospital visits, and how this had prevented regular family activities. Siblings were often not included in hospital visitation and had to learn to look after themselves at a young age in an empty house. Feeling almost like they were living separate lives, parents concurred that they had lost valuable time with their other children (eg, Table 3, quote 8). With the relative with IBD becoming the parents’ main focus, siblings could feel jealous or marginalized, and that they were a lower priority. This constant hyperfocus on supporting the person with IBD also meant that siblings believed their own achievements were diminished in the eyes of their family (eg, Table 3, quote 9).

#### Subtheme 4: no relationship is easy

Despite the negative impact of the disease on the family, almost all of the participants also expressed how family bonds had strengthened after the diagnosis of a family member with IBD. Working together to support each other provided them with a shared sense of resilience (eg, Table 3, quote 10). However, partners of people with IBD spoke about how maintaining intimacy was often difficult due to symptoms and emotional issues. Family members also explained how sometimes they clashed with their relatives with IBD in terms of the best ways to control their symptoms (eg, Table 3, quote 11). Not only did the functional tasks of caring affect relationships, but also the strain of worrying about the person whom they cared for was detrimental to relationships between other family members (eg, Table 3, quote 12).

### Theme 2: There Have Been a Lot of Bridges to Cross Along the Way

Several varied coping strategies were employed by families to cope with the impact of IBD, and many participants reported using several different types of coping to manage the situations arising:

“I think my coping mechanism actually probably is to make sure that my daughter is getting the right treatment for her disease, and I think that’s how I cope. If I’m stressed, I talk to my husband about it, or I talk to my friends.” (Family13, FM22, Mother)

#### Subtheme 1: powerful social support network

Accessing emotional support, open communication, and seeking help with household chores were common approaches to minimizing stress levels within the family. For some relatives, friends were a “safe zone” to talk about their worries without upsetting their relatives. Members of the extended family and friends were also drawn upon for both practical support such as childcare and emotional support at crisis moments or just to provide respite (eg, Table 3, quote 13). Participants also described how IBD charities were key to staying informed about the disorder and obtaining practical knowledge which helped reduce their stress. Learning from others’ experiences of living with IBD and accessing self-help groups were examples of proactive coping strategies (eg, Table 3, quote 14). Participants who had to balance their full-time jobs with their caregiver roles also mentioned that understanding and empathy from their employer and colleagues were highly valued (eg, Table 3, quote 15).

#### Subtheme 2: putting your head in the sand

Participants explained how they sometimes tried to avoid difficult situations rather than tackle issues head-on. Sometimes they tried to distract themselves, even in a crisis, by immersing themselves in their everyday lives (eg, Table 3, quote 16). However, while a few family members mentioned that this strategy could be helpful at the time, it was not always seen as a viable long-term solution. Some patients and family members reported using alcohol to help relieve stress and pressure. In some cases, alcohol use became problematic, which again affected the emotional well-being of other relatives (eg, Table 3, quote 17).

#### Subtheme 3: acceptance and moving on

Participants also spoke about how cultivating acceptance of the situation could help them to adapt to life with IBD. Realizing that there could be issues ahead and considering them in advance to try and minimize the impact of the disease was a key approach to reducing stress and maintaining resilience. People spoke about planning carefully for holidays and trips to minimize disruption (eg, Table 3, quote 18).

Using humor, such as crafting jests related to symptoms of IBD, also relieved tension for some participants. Equally, participants referred to “looking at the bright side” or trying to be more optimistic about life generally (eg, Table 3, quote 19). Some participants also mentioned using uplifting activities such as exercise, meditation, yoga, and playing with their pets to relieve stress. When they were able to do these types of activities together as a family, it strengthened the bonds between them and helped everyone’s well-being (eg, Table 3, quote 20).

### Theme 3: My Life Would Be Better if…

To mitigate the negative impact of IBD, participants expressed the need for greater support for carers and wider family members. This included proactive and effective communication from healthcare professionals (HCPs), along with referrals to sources of information, therapeutic services, and support groups.

#### Subtheme 1: You listen to me and talk to me

HCPs’ failure to consider and include family members as part of care (ie, during care planning, consultation, and routine care delivery) was considered a serious oversight by participants. They needed clear, meaningful, and honest communication about their relative’s condition, and the future implications for them and their families. Access to advice or support was especially important during the stressful prediagnosis and initial diagnosis period (eg, Table 3, quote 21).

Indeed, many participants expressed the need for an identified liaison person to communicate with family members about the disease and treatments, or the implementation of a helpline to connect professionals and family members (eg, Table 3, quote 22). Even though many family members wanted to know more about the disease and treatment of a patient, they were aware that confidentiality and privacy issues would sometimes make this difficult, especially with adult patients (eg, Table 3, quote 23).

#### Subtheme 2: giving me better information

At all points in the patient’s disease trajectory, caregivers wanted accurate and useful information, communicated in lay language, on the course of the disease, the care required, and treatment demands. Participants also described that in some cases, child-friendly versions of some IBD-related resources would be helpful. There was also a request for greater information on how to live normally with IBD, and specifically a greater explanation of maintaining intimacy and fertility implications which could be shared with partners (eg, Table 3, quote 24).

Family members often felt unprepared to provide IBD care, and received little guidance from HCPs on how to access appropriate resources (eg, Table 3, quote 25). Leaflets, booklets, face-to-face information from a specialist, and online platforms were mentioned as potentially useful ways to deliver this information. Information provided on the website managed by Crohn’s and Colitis UK was found to be helpful and accessible. However, without being directed to appropriate websites, relatives could become overwhelmed by the amount of information online and anxious about the content of some sites (eg, Table 3, quote 26).

#### Subtheme 3: provide me with meaningful psychosocial support sources

Despite the significant burden of caring for and living with people with IBD, participants perceived a distinct lack of interest by health or social care organizations in helping families. Many felt unsupported as they tried to take on new caregiver roles, adjust family life, and grapple with lower incomes and greater costs (eg, Table 3, quote 27). The strain of caring meant that family members sometimes neglected their own mental health. This was particularly problematic for those participants who already had their own emotional issues before their relative’s diagnosis. There was enthusiasm for the provision of individual and family counseling centered specifically around IBD, and for the opportunity to connect with peers who were also caregivers and relatives of people with IBD to share experiences (eg, Table 3, quote 28).

## Discussion

### Main Outcomes

The aim of this study was to understand the experiences of families who support an adult family member with IBD. Proponents of FST state that the health of an individual family member can have a profound impact on the well-being of other members within the family unit. This can result in challenges for family members as they strive to manage and adapt to the impact of an illness on their lives.^24^ Findings from this study certainly highlight the impact of IBD not only on the main caregivers or partners, but also across the wider family unit. Parents in this study reported anxiety around the future health of their child and the strain of juggling caring duties with the needs of the rest of the family and other children.

In particular, the largely negative experiences of the siblings of the person with IBD were of note. Strong feelings of isolation in their formative years, as a result of their parents’ continual visits to hospital, were also expressed by siblings. A previous study exploring the experiences of parents and siblings of children with IBD also found not only fear regarding the health of their brother or sister, but also emerging feelings of resentment and jealousy.^26^ The unpredictable nature of IBD placed considerable limitations on family plans for travel, hobbies, and social lives, which had had a significant impact on other children in the family. A survey by Becker et al^27^ found that over a third of family members reported having less time for hobbies and private time.

As with most chronic diseases, the time immediately after diagnosis is particularly difficult for patients and for their family members.^28^ Previous IBD studies have shown that initially adapting to an unfamiliar disease and its management can cause a stressful period for family members.^14^ The findings of the current study reaffirm previous researchers’ observations that family members are uncertain as to what to expect in the future, and worry about their relative dying.^15,29^ The presence of a familial IBD history is one of the identifiable risk factors for IBD development; this fear and worry sometimes extended to their own health, which is unsurprising given the increased risk of the disorder in first-degree relatives.^30^

This study confirmed that the emotional effects of living with and caring for a relative with IBD can be intense, and can include frustration, anxiety, and sadness. Previous studies also showed that caring for a family member with IBD substantially lowers the QoL of other family members.^11,15,27^ In particular, the episodic and unpredictable nature of the condition itself, with severe flare-ups and then periods of relative stability, is commonly reported as a source of stress in families and affects the ability to plan.^13^

Participants in this study relayed how a diagnosis of IBD required them to take on new roles or responsibilities, such as providing physical care and financial and emotional support. Our findings support other studies indicating that medication, hospital visits, and special diets can place a considerable financial strain on the family.^31^ Some members of the family had to undertake additional employment or extend their working hours to provide financial support for their families. Participants reported low productivity, absences, and working part-time to manage their carer roles, which were also reported by other studies looking at the financial impact of IBD on the family.^32^ These additional responsibilities put relationships under strain.^18^ Indeed, conceptual models of caregiver burden in chronic disease propose that the stress and burden experienced by caregivers are not solely attributed to the care recipient’s health, but also attributed to financial difficulties, work-related stress, and the caregiver’s health.^33^

It is common for families caring for a person with a chronic disease to feel closer, and to undergo a bonding experience as a result of the illness and caring journey.^28^ This study also found that the family members believed that IBD had made them “tougher,” more open about illness, and more aware of disability generally. Research suggests that families strive to maintain balanced functioning or homeostasis by utilizing their available resources and coping strategies.^34^ However, as shown in this study and others, families living with chronic disease may use maladaptive coping strategies such as denial, avoidance, self-blame, and substance use, which tend to increase family distress and decrease QoL.^35,36^

This study also demonstrated that participants felt more in control when they were able to use proactive approaches such as seeking social support, planning in advance, employing humor and hope in daily life, and investing in well-being activities such as yoga as a family. Such coping strategies are thought to be one of the key components of family resilience.^37^ HCPs can help families to build this resilience by promoting adaptive coping strategies such as reframing or acceptance, in addition to helping to address any maladaptive coping strategies, such as denial or alcohol use.

Caregivers who can cope effectively with the challenges of caregiving will be better equipped to support the patient and improve their health outcomes.^18^ Families commonly wanted a better understanding of their relative’s condition and how to optimally support them. However, the feelings of participants in this study reflect other caregivers of people with IBD, who commonly report^14^ dissatisfaction with the communication they receive from professionals, and the information and resources available for them, particularly family members.^14^

Despite the high levels of stress and anxiety reported by participants, few family members had accessed support for their own well-being. Sometimes this appeared to be due to insufficient time, a lack of access to services, or feeling guilty that they needed help when not “unwell.” Such feelings of guilt have been recorded in previous studies of IBD partners.^16^ However, on the whole, participants felt that individual or family counseling centered specifically around IBD would be beneficial. As in previous studies,^38^ some were also keen to reach out to peer support groups. Research suggests that such groups which include education and supportive discussion can reduce the burden of IBD experienced by families,^12^ improve knowledge, and facilitate adaptive coping mechanisms within the family unit.^39,40^

### Practice Recommendations

Given the burden experienced by the family when a member has IBD, it is important that healthcare services understand the importance of adopting a collaborative approach that involves patients, family members, and HCPs. Indeed, as the involvement of caregivers is likely to be of benefit to the patient this is central to providing patient-centered care. This approach is also advocated in the IBD UK Standards of Care (2019), which recommend that professionals should actively involve family members in their relative’s care, where appropriate and with the patient’s consent.^17^ There is also clearly a desire by family members to meet with others who live with people with IBD to share their experiences and get practical information. This highlights the potential for peer support groups.

IBD treatment should also be holistic and delivered by multidisciplinary teams that include medical staff, IBD nurses, counselors or psychologists, and social workers. In addition to maximizing the quality of clinical care, a holistic approach should encompass the wider social, emotional, and financial support that might be needed by family members to maintain resilience, and to maximize their ability to provide good quality care for their loved ones. Equally, it is important to listen to the siblings of people with IBD and to provide tailored support to help minimize feelings of isolation or loneliness during their relatives’ IBD flare-ups. In the United Kingdom, it is important that services for IBD meet the IBD Standards of Care (2019), which recommend offering options for counseling or referral to specialist services where appropriate to patients and their family members.^17^

### Strengths, Limitations, and Implications for Future Research

This is the first research exploring the impact of IBD on family members in the United Kingdom. The sample represented a range of kinship relations, including parents, partners, siblings, children, and family members’ experiences at different stages of the disease. However, the majority of participants identified themselves as White British, which reflected the membership demographics of the charities from whose social media networks they were recruited. However, the impact of IBD on family members is likely to be experienced differently by individuals from other cultural groups. It is important to explore how burden and support are experienced by families from different cultures to ensure services are tailored to their needs.

This study aimed to capture a comprehensive spectrum of family experiences with IBD, and intentionally did not specify a time frame cohabitation duration for inclusion (ie, we did not study the variable of how long family members had lived with the person with IBD). While this approach broadened the research scope, it may have introduced unintended heterogeneity related to exposure to the impact of IBD. Therefore, the lack of data on cohabitation length hinders our ability to fully account for this potential moderating factor and limits the generalizability of findings. Future research in this area would benefit from specifying cohabitation duration and capturing detailed cohabitation history to enable a more nuanced understanding of how varying exposure to IBD interacts with family dynamics and outcomes.

Conducting interviews at a single time point cannot comprehensively capture the full complexity and evolution of participants’ thoughts, emotions, and experiences over the trajectory of their experience. Furthermore, participants’ recollections of past events or experiences may be subject to memory biases. In future research, it will be important to conduct larger, longitudinal studies, which can be used to explore how the severity and relapses of IBD can impact the family to understand how best to provide support at crisis points. Given the current dearth of family interventions for people with IBD,^13^ there is a pressing need to develop such interventions.

## Conclusions

IBD can have a significant impact not only on the individual who has the condition, but also on their family members. HCPs can support family members by employing proactive communication, providing appropriately targeted information, offering emotional support, and promoting family collaboration. It is important for HCPs to consider the coping strategies used by families and promote resilience and adaptation among family members.

## Supplementary data

Supplementary data is available at *Inflammatory Bowel Diseases* online.

izae028_suppl_Supplementary_Material

## Data Availability

The data underlying this article cannot be shared publicly due to the sensitive nature of the research and the privacy of individuals that participated in this study.
