# Supplementary material for: Our Life Is a Rollercoaster! A Qualitative Phenomenological Study Exploring the Impact of IBD on Family Members
Source: Inflamm Bowel Dis. 2024 Feb 28;30(12):2395–404. doi: 10.1093/ibd/izae028 (PMC11630016; doi:10.1093/ibd/izae028)
Supplement: izae028_suppl_Supplementary_Material [file izae028_suppl_supplementary_material.docx]

**Tables**

*Table 1: Topic guide for semi-structured interview (patient)*

| **Opening question** | **Can you tell me about your experience of having IBD and how this affects your life?** |
| --- | --- |
|  | - How did you feel when you were diagnosed with IBD? - Were you admitted to hospital in relation to your IBD? - Can you briefly tell me about your symptoms of IBD since diagnosis until now? - Can you tell me if having IBD impacts your life and how?   Prompt: - Relationship/s in family  -Relationship with others (friends/ colleagues/…)  -Psychological wellbeing (fear/guilt/…)  -Everyday life (financial/leisure time/travel/work/...) |
| **Explorative question** | **Please can you tell me if you think you having IBD impacts your family members?** |
|  | - What were your family members’ reactions to you being diagnosed with IBD? - Do you think being diagnosed /having IBD has affected your family? - Can you tell me who are the members of your family and how have they been affected by your IBD/ by you having IBD? - Explore the impact of IBD on different family members and the impact on the whole family.   To use prompts to explore the impact of IBD on different family members:  - Relationship/s in family  -Relationship with others (friends/ colleagues/…)  -Psychological wellbeing (fear/guilt/…)  -Everyday life (financial/leisure time/travel/work/..)   - To use reflective questioning to explore the topic that the participant brings up, e.g. How do you feel about ………? Can you tell me more about it? |
| **Follow up question** | **Do you have any ways of making your life and that of your family easier?** |
|  | - Do you discuss the impact of IBD on your family with other  family members?   - What did you or your family members do to mitigate the impacts of IBD on their lives? (What methods and strategies have you and your family members tried?) - Did you and your family members receive any help or support from outside family e.g. the support to cope with IBD or information support, and if so, what type of help was it? - How beneficial and effective where the support systems for your family members? - Ask reflective and clarifying questions to find out information on the type of help and support received and their effectiveness. |
| **Follow up question** | **Help and support needed** |
|  | - Are there particular problems in your family related to IBD that you feel need better support? - What help and support your family member/s needed that wasn’t available at the time? - What help and support would your family members need now? - What advice would you give to the newly diagnosed patients and their families to adapt to living with IBD better? - Use reflective questioning to explore the topic further. |
| **Closing question** | **Is there anything else that you would like to add?** |

*Table 2: Topic guide for semi-structured interview (family members)*

| **Opening question** | **Please can you tell me how (named person or relationship) having IBD impacts your life?** |
| --- | --- |
|  | - How did you feel about (patient name) being diagnosed with IBD? - Tell me about your life since (patient name) was diagnosed with IBD? How has your life been affected?   Prompt: - Relationship/s in family  - Relationship with others (friends/ colleagues/…)  - Psychological wellbeing (fear/guilt/…)  - Everyday life (financial/leisure time/travel/work/..)  -How do you feel about …………?  (use reflective questioning to explore the topic further) |
| **Explorative question** | **Do you have any ways of making life with IBD easier in your**  **family?** |
|  | - Do you discuss the impact of IBD on the family with other family members?   - What did you do to mitigate the impacts of IBD on you? - What did you do to mitigate the impacts of IBD on other family members? - How effective were these methods? - What other help and support did you have at the time? e.g. the support to cope with IBD or information support, and if so, what type of help was it? - What help and support did you need that wasn’t available at the time? - (Use reflective and exploratory questions to find more information and clarification) |
| **Follow up question** | **Help and support needed** |
|  | - Are there particular problems in your family related to IBD that you feel need better support? - What help and support do you needed that wasn’t available at the time? - What help and support do you need now? - What advice would you give to the newly diagnosed patients and their families to adapt to living with IBD better? - Use reflective questioning to explore topic features. |
| **Closing question** | **Is there anything else that you would like to add?** |
